# Transcatheter Ventricular Septal Defect (VSD) Creation for Restrictive VSD in Double-Outlet Right Ventricle

**DOI:** 10.1007/s00246-012-0337-1

**Published:** 2012-05-12

**Authors:** C. Huie Lin, Charles Huddleston, David T. Balzer

**Affiliations:** 1 Division of Pediatric Cardiology, Department of Pediatrics, Washington University School of Medicine, 8th Floor NWT, 1 Children’s Place, St. Louis, MO 63110 USA; 2 Department of Surgery, SSM Cardinal Glennon Children’s Medical Center, St. Louis, MO USA

**Keywords:** Restrictive VSD, Transcatheter VSD creation, VSD stenting

## Abstract

**Background:**

Double-outlet right ventricle (DORV) with a restrictive ventricular septum is a rare but highly morbid phenomenon that can be complicated by progressive left ventricular hypertrophy, arrhythmias, aneurysm formation, severe pulmonary hypertension, and death in the newborn. Surgical creation or enlargement of a ventricular septal defect (VSD) is palliative but may damage the conduction system or the atrioventricular valves in the newborn. This report presents a transcatheter approach to palliation for a newborn that had DORV with a restrictive ventricular septum.

**Methods/Results:**

A full-term infant girl (2.9 kg) referred for hypoxia (80 % with room air) and murmur was found to have DORV, interrupted inferior vena cava, and restrictive VSD (95-mmHg gradient). Transhepatic access was performed, and an internal mammary (IM) catheter was advanced through the atrial septal defect and into the left ventricle. By transesophageal echocardiographic guidance, a Baylis radiofrequency perforation wire was used to cross the ventricular septum, and the defect was enlarged using a 4-mm cutting balloon. A bare metal stent then was deployed to maintain the newly created VSD. The patient did well after the procedure but required pulmonary artery banding 4 days later. She returned 5 months later with cyanosis and the development of obstructing right ventricle muscle bundles, requiring further surgical palliation.

**Conclusions:**

This report describes the first transcatheter creation of VSD in DORV with a restrictive ventricular septum in a newborn infant. Use of the radiofrequency catheter in combination with cutting balloon dilation and stent implantation is an efficient method for creating a VSD in such a patient.

**Electronic supplementary material:**

The online version of this article (doi:10.1007/s00246-012-0337-1) contains supplementary material, which is available to authorized users.

## Introduction

A restrictive ventricular septal defect (VSD) in the setting of a double-outlet right ventricle (DORV) is a rarely reported phenomenon that can lead to complications including progressive left ventricular (LV) hypertrophy, arrhythmias, aneurysm formation, severe pulmonary hypertension (with an intact or restrictive atrial septum), and death [1–6, 8, 9]. Progression of the LV outflow obstruction to complete atresia has been reported [5]. Surgical enlargement of the native VSD may damage the atrioventricular (AV) valves or conduction tissue. Transcatheter VSD creation or enlargement to address this issue has rarely been reported [6]. We report transcatheter VSD creation in a unique case of DORV with a restrictive VSD in a patient not amenable to a biventricular repair. To our knowledge, this is the youngest, smallest patient reported to date.

## Case Report

The girl in this case was born via repeat Cesarean section after a full-term gestation. Found to have oxygen saturations in the 80’s on room air as well as a heart murmur, she was referred to our institution for cardiac evaluation and treatment.

Initial examination demonstrated a nondysmorphic child weighing 2.9 kg. Her oxygen saturations with room air were 90 %, and her cardiac examination demonstrated a normal S1 and S2. A harsh grade 3 systolic ejection murmur was best heard at the left upper sternal border. The diastole was quiet. Her pulses were full and equal in the upper and lower extremities.

An echocardiogram demonstrated atrial situs solitus. There was a DORV with d-malposition of the great vessels and mild subpulmonary obstruction with an estimated gradient of 20 mmHg. The inferior vena cava (IVC) was interrupted with azygous continuation to a right-sided superior vena cava. There was LV hypertrophy with a very restrictive remote (noncommitted) VSD (Supplementary Video 1). The gradient across the VSD was 95 mmHg, and the defect appeared to be filled with accessory AV valve tissue. The atrial septal defect was moderate in size, and there was no evidence of mitral stenosis.

Surgical decompression of the LV was thought to be of high risk for AV valve damage or complete heart block. Therefore, the patient was sent for transcatheter VSD creation at the age of 17 days.

The procedure was performed with the patient under general anesthesia. Because of the interrupted IVC, transhepatic access was obtained, and a 5-Fr sheath was placed. A 5-Fr sheath also was placed in the right internal jugular vein. A diagnostic catheterization was performed, which documented the aforementioned anatomy and demonstrated an LV systolic pressure of 128 mmHg and a right ventricle (RV) systolic pressure of 53 mmHg. There was subpulmonary obstruction with distal pulmonary artery (PA) pressures of 21/11 mmHg. The Qp/Qs ratio was 4.25:1.

**Fig. 1:**
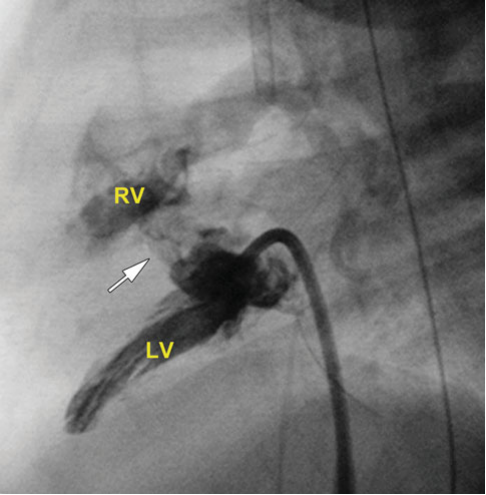
Lateral view of the left ventriculogram demonstrating a restrictive ventricular septal defect (VSD) (*white arrow*). *RV* right ventricle, *LV* left ventricle

An LV gram demonstrated the restrictive VSD (Fig. 1). The atrial septum was crossed via the transhepatic access, and a 4-Fr internal mammary (IM) catheter (Cordis Corp., Miami Lakes, FL, USA) was advanced over a guidewire into the LV. In combination with fluoroscopy and angiography, transesophageal echocardiography (TEE) was used to guide the procedure, allowing positioning of the IM catheter against the muscular septum. A Baylis radiofrequency (RF) perforation wire (Baylis Medical Co., Montreal, Canada) was placed through the coaxial catheter, and these were then advanced to the catheter tip. Radiofrequency energy was applied using 10 W for 2 s. The wire easily crossed the septum. The coaxial microcatheter then was advanced over the Baylis wire into the RV. The Baylis wire was removed, and initially, a 0.018-in. Terumo Glidewire (Terumo Corp., Tokyo, Japan) was advanced into the RV. The wire was noted to cross the free wall of the RV and enter the pericardial space. A small pericardial effusion associated with hypotension and ventricular dysfunction was noted. The wire was removed, and emergent pericardiocentesis was performed, with 12 ml of blood removed through a 5-Fr pigtail catheter placed in the pericardial space. This resulted in significant hemodynamic improvement.

**Fig. 2:**
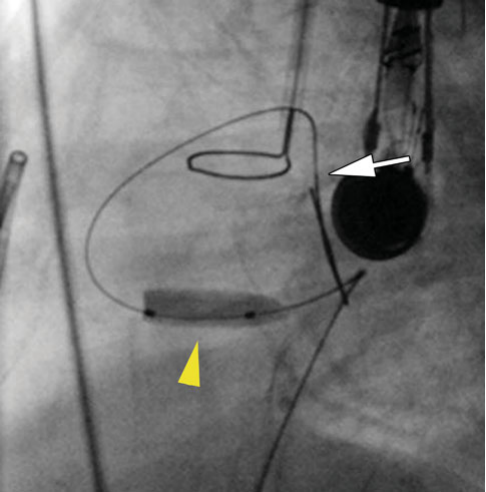
Lateral view of the guidewire that has been advanced from the transhepatic approach across the newly created ventricular septal defect (VSD). The tip of the guidewire is in the pulmonary artery (*white arrow*), and a cutting balloon (*yellow arrowhead*) is inflated across the ventricular septum

After hemodynamic stabilization, the ventricular septum was crossed again using the Baylis wire. However, this time, a 0.014-in. coronary guidewire was advanced across the septum. The wire could not be manipulated into the PA or the aorta, so a continuous wire loop was not achieved. A 4-mm Flextome cutting balloon (Boston Scientific, Natick, MA, USA) then was advanced over the wire across the septum and inflated (Fig. 2). The cutting balloon was removed, and a 4-Fr multipurpose coronary catheter (MPA) (Cordis Corp.) catheter was advanced across the newly created VSD. A 0.035-in. Rosen wire (Cook, Bloomington, IN, USA) then was advanced through the MPA catheter into the RV, and the MPA catheter was removed. The transhepatic sheath was exchanged for a 6-Fr sheath, and a PG1280 BPX stent (Cordis Corp.) was advanced over the Rosen wire to cross the septum.

**Fig. 3:**
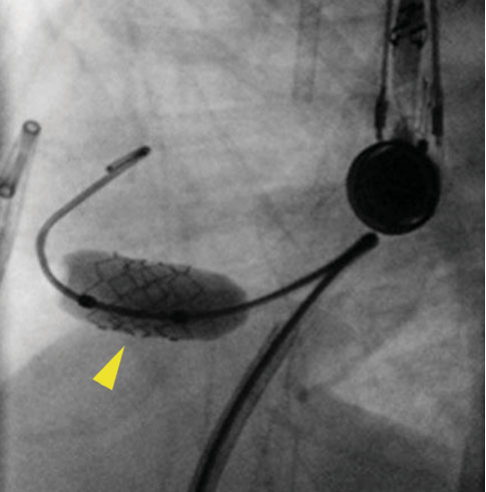
Lateral view of the stent (*yellow arrowhead*) being deployed across the ventricular septum

**Fig. 4:**
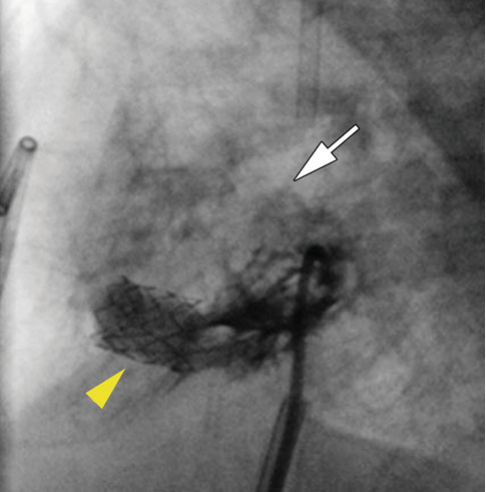
Lateral view of the left ventriculogram after stent deployment demonstrating flow across the newly created ventricular septal defect (VSD) (*yellow arrowhead*) and the relatively small flow across the native VSD (*white arrow*)

To guide proper placement of the stent, TEE guidance was used. Once the stent was in a good position, the balloon was inflated, and the stent was deployed (Fig. 3). Follow-up angiography and TEE demonstrated that the stent was in excellent position (Fig. 4). After stenting, the systolic LV pressure fell to 83 mmHg, with a cuff pressure of 65 mmHg.

The immediate postintervention course was relatively uncomplicated (Supplementary Video 2). The patient was noted to have occasional runs of monomorphic ventricular tachycardia, which were not hemodynamically compromising. She was initially administered a beta-blocker but continued to have ectopy, so amiodarone was initiated, which resolved her ectopy. However, 4 days after the interventional procedure, the patient had persistently high oxygen saturations, only mild subpulmonic obstruction, and a high QP/QS with repeat catheterization. As a result, the she was referred for surgical PA banding 4 days later. Her postoperative course was unremarkable, and she was discharged home after an uneventful recovery.

**Fig. 5:**
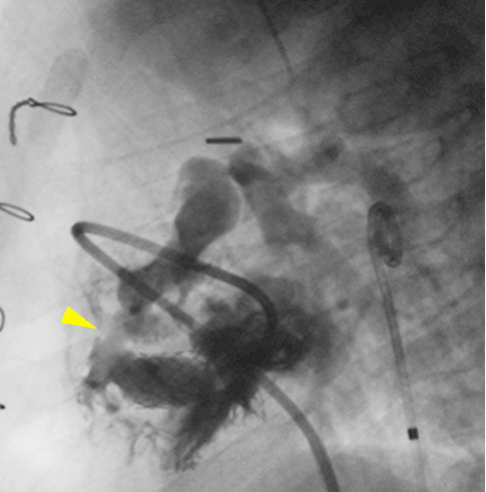
Left ventriculogram demonstrating the development of the obstructing muscle bundles (*yellow arrowhead*) on the right ventricular (RV) side of the ventricular septal defect (VSD) stent and in the subpulmonary region

The patient did well initially but returned at the age of 5 months with increasing cyanosis. A diagnostic catheterization demonstrated a very tight PA band with aortic saturations of 50 % and a Qp/Qs of 0.38. She had experienced interval obstruction of the stented VSD secondary to muscle bundles within the RV at the end of the stent, resulting in a 54-mmHg gradient across the VSD (Fig. 5). She was taken to the operating room 5 days after the catheterization, where the obstructing RV muscle bundles and the PA band were removed, the PA was oversewn, and a pulmonary valvectomy was performed. Because of her small size and the presence of an interrupted IVC, a Glenn shunt was not placed. To provide pulmonary blood flow, a 6-mm PTFE graft (W.L. Gore, Flagstaff, AZ, USA) was sewn from the RV to the PA. The patient did well postoperatively and at this writing is awaiting further palliation. She has had no further ventricular ectopy, and the amiodarone has been discontinued.

## Discussion

A restrictive VSD with DORV is a rarely reported phenomenon. The fate of the hypertensive LV is not well defined in this circumstance, but progressive LV hypertrophy, LV dysfunction, arrhythmias, ventricular aneurysms, myocardial fibrosis, and progression to complete VSD occlusion with resulting death all have been reported [1–6, 8, 9]. Given the rarity of this lesion, the optimal management strategy remains undefined. Surgical approaches have included atrial septectomy and either mitral valve avulsion [10] or LV exclusion by suturing of the mitral valve and sewing of a patch in the supra-annular position [7]. Surgical decompression of the hypertensive LV by enlargement or creation of a VSD also can be considered, but there is risk to the conduction system and the AV valves with this approach.

Ideally, VSD creation in the mid-muscular septum using transcatheter methods could avoid these potential complications. Meadows et al. [6] reported their results with transcatheter VSD creation or enlargement in eight patients ranging in age from 9 months to 15 years. The procedure was acutely successful for all the patients, but recurrent obstruction developed in the majority. Two patients experienced AV valvular regurgitation, and one patient required surgical repair.

We report a patient in whom a VSD was created percutaneously to highlight further the results of this approach. From a technical standpoint, the reported patient is unique in that she had an interrupted IVC, necessitating a transhepatic approach for VSD creation because we wanted to create the VSD from an LV approach. The transhepatic approach also was advantageous because the patient weighed only 2.9 kg at the time of the initial procedure. Radiofrequency perforation of the septum was successful for the reported patient in crossing of the septum, as opposed to Meadows et al. [6], for whom this technique was unsuccessful on two occasions.

The reported patient did experience a myocardial perforation with a pericardial effusion. This resulted not from the RF wire passage but rather during passage of the 0.018-in. Terumo Glidewire. This likely resulted from the microcatheter abutting the RV free wall as the guidewire was passed and should be an avoidable complication. Meadows et al. [6] had no cases of perforation during their procedures.

The reported patient also experienced recurrent obstruction of the stented VSD. Similar to the experience of Meadows et al. [6], the obstruction occurred at the RV end of the stent and was secondary to progressive hypertrophy of trabeculations or muscle bundles within the body of the RV. Meadows et al. [6] hypothesized that this could be avoided with complete relief of the obstruction at the time of the initial VSD creation. This could theoretically avoid LV hypertension and progressive ventricular hypertrophy.

The reported patient had a residual 18-mm gradient at the time of her initial VSD creation. In addition, she had a PA band placed 4 days after the VSD creation. This likely led to progressive RV hypertrophy and recurrent VSD obstruction.

The optimal management strategy for DORV with restrictive VSD remains unknown. Relief of LV hypertension is mandatory for the reasons delineated earlier, and both surgical and, more recently, transcatheter approaches are feasible. Long-term outcomes still are poorly defined, so no definitive recommendations can be made concerning which approach is best.

## Electronic supplementary material

Below is the link to the electronic supplementary material.

Video 1 Two-dimensional and color-Doppler transthoracic echocardiography before the procedure. The parasternal long-axis view demonstrates the restrictive native ventricular septal defect (VSD) with accessory atrioventricular (AV) valvular tissue partially occluding the defect. (AVI 2700 kb)

Video 2 Two-dimensional and color-Doppler transthoracic echocardiography after successful intervention. The newly placed ventricular septal defect (VSD) with the stent placed can be seen and is widely patent with a brisk flow on color-Doppler. (AVI 2625 kb)
